# Commentary on the article “Postpartum women’s use of medicines and breastfeeding practices: a systematic review”

**DOI:** 10.1186/s13006-016-0080-y

**Published:** 2016-07-21

**Authors:** Laurence Spiesser-Robelet, Rémi Gagnayre

**Affiliations:** Laboratory Education and Health Practices (EA 3412), Université Paris 13, Sorbonne Paris Cité, Bobigny, France; Pharmacy Department, Angers University Hospital, Angers, France

**Keywords:** Breastfeeding, Lactation, Medication, Mothers’ behavior

## Abstract

This commentary follows the article of Moni R. Saha and her co-authors, entitled *"Postpartum women's use of medicines and breastfeeding practices: a systematic review."* As highlighted in this systematic review, medication use is common during the postpartum period often creating difficulty for mothers. Several studies illustrate the negative impact medication has on breastfeeding, initiation and duration despite reassuring advice from health professionals. Current data only describe the use of medication and behavior adopted by mothers when medication is prescribed. The factors influencing maternal behaviors have not been studied. Behaviors depend on knowledge, representations and attitudes. To better understand the behaviors of mothers faced with medication, we conducted a qualitative study, utilizing semi-structured interviews to investigate knowledge, risk perception and difficulties women experienced. The study consisted of a description and comparison of the perceived needs of two populations: 19 breastfeeding mothers and 12 health professionals. Divergences between the two populations were highlighted, focusing specifically, on knowledge needed by the women. This commentary is intended to highlight the need for further research essential to explain the influences on maternal behavior when medication is a consideration, allowing health professionals to better help mothers deal with these situations frequently affecting their breastfeeding plans.

## Main text

We read with great interest the article of Moni R. Saha and her co-authors, entitled *"Postpartum women's use of medicines and breastfeeding practices: a systematic review"*, which assesses the extent of drug use in women postpartum but also the impact of these drugs on the initiation and duration of breastfeeding [1].

Breastfeeding is a real public health issue in terms of its benefits for children as well as mothers [2–4]. The question of drug use is a sensitive point. It refers to several findings that may seem contradictory. On one hand, mothers describe a drug as a reason to stop breastfeeding [5, 6]. On the other hand, biomedical data, even if they are few, indicate that most drugs are excreted in breast milk but in small quantities and are rarely the cause of serious adverse events for the newborn [7, 8]. Finally, taking medication is not an isolated or occasional phenomenon; Saha et al. indicate that over 50 % of women face this situation during the postpartum period [1]. These data are confirmed by Howard and Lawrence's study of more than 14,000 women in the United States [9]. They found that more than 79 % of breastfeeding women were faced with having to take at least one medication while nursing (3.3 different drugs on average) [9].

As Saha et al. described, despite limited data (5 studies, small samples, often specific drugs), the drug has a negative impact on the initiation and duration of breastfeeding. But no data are available on *"how women make decisions on that subject and the factors influencing them?*" [1]. It seems interesting to highlight issues that were not discussed. On one hand, women request an opinion of the health professionals about the compatibility of treatment with breastfeeding in several studies [10–12]. On the other hand, non-initiation or weaning during treatment in other words refusal to take medication and continuing breastfeeding are often choices women feel they must make, despite the reassuring advice of health professionals [10–12]. This discrepancy makes us wonder about the influences affecting maternal behaviors.

This review, in addition to the publications *by* Hussainy et al. and McDonald et al. [13, 14], concluded that research should be developed to understand these phenomena. In fact, studies on medication and breastfeeding describe which medications are taken during breastfeeding and also the mothers’ behavior, i.e. the way they act in these situations. In view of these results regarding the negative impact of drugs on breastfeeding, it is essential to understand what influences mothers’ behavior, when medication is prescribed. Behaviors depend on knowledge, representations and attitudes [15–19].

In a study entitled *"Analysis of breastfeeding mother's needs towards medication use"* we explored the knowledge of mothers about medication, understanding of risks associated with drugs and the mothers’ difficulties related to medication use while breastfeeding, from the perspective of 19 breastfeeding mothers and 12 health professionals [20]. Analysis of the interviews with the women showed low levels of knowledge about how drugs are excreted in human milk. The women also describe anxiety and feelings of guilt. Six of the 19 women stopped breastfeeding because of the medication. The main educational needs expressed both by the women and the health professionals were: to know the suitable treatment for current diseases; to understand how drugs are excreted in human milk; and finally questions which must be considered before taking medication. Divergences between the breastfeeding women and the health professionals regarding necessary knowledge were highlighted. Women expressed the need to understand the possible consequences of their treatments on their child, while the professionals believed this information should be limited so as to not worry mothers. These results show the difficulties as health professionals and mothers do not always have the same perception of breastfeeding women’s needs. They also highlight an ambiguity women face believing that some drugs can be used during breastfeeding yet with other drugs, refusing to take medication or halting breastfeeding in order to take medication. This first study was conducted on a small number of women and health professionals [20]. These results are preliminary. Our results must be confirmed and deepened by other studies.

In view of these data, future research should aim to understand rather than describe mothers’ behaviors when faced with medication use. The major issue is to help to prevent failure of breastfeeding and to implement educational actions empowering women facing these particular difficulties.
